# Direct comparison study of DNA methylation markers in EpCAM-positive circulating tumour cells, corresponding circulating tumour DNA, and paired primary tumours in breast cancer

**DOI:** 10.18632/oncotarget.18679

**Published:** 2017-06-27

**Authors:** Maria Chimonidou, Areti Strati, Nikos Malamos, Sophia Kouneli, Vassilis Georgoulias, Evi Lianidou

**Affiliations:** ^1^ Analysis of Circulating Tumour Cells Laboratory, Laboratory of Analytical Chemistry, Department of Chemistry, University of Athens, Athens, Greece; ^2^ Department of Pathology, Oncology Unit, Helena Venizelou Hospital, Athens, Greece; ^3^ Laboratory of Tumour Cell Biology, Medical School, University of Crete, Heraklion, Greece

**Keywords:** liquid biopsy, circulating tumour cells, circulating tumour DNA, breast cancer, methylation specific PCR

## Abstract

Circulating Tumour Cells (CTCs) and circulating tumour DNA (ctDNA) represent a non-invasive liquid biopsy approach for the follow-up and therapy management of cancer patients. We evaluated whether DNA methylation status in CTCs and ctDNA is comparable and whether it reflects the status of primary tumours. We compared the methylation status of three genes, SOX17, CST6 and BRMS1 in primary tumours, corresponding CTCs and ctDNA in 153 breast cancer patients and healthy individuals, by using real time methylation specific PCR. We report a clear association between the EpCAM-positive CTC-fraction and ctDNA for SOX17 promoter methylation both for patients with early (*P* = 0.001) and metastatic breast cancer (*P* = 0.046) but not for CST6 and BRMS1. In early breast cancer, SOX17 promoter methylation in the EpCAM-positive CTC-fraction was associated with CK-19 mRNA expression (*P* = 0.006) and worse overall survival (OS) (*P* = 0.044). In the metastatic setting SOX17 promoter methylation in ctDNA was highly correlated with CK-19 (*P* = 0.04) and worse OS (*Ρ* = 0.016). SOX17 methylation status in CTCs and ctDNA was comparable and was associated with CK-19 expression but was not reflecting the status of primary tumours in breast cancer. DNA methylation analysis of SOX17 in CTCs and matched ctDNA provides significant prognostic value.

## INTRODUCTION

During the last years, intensive basic research has led to an increasing number of treatment options for cancer patients. This in turn has created an urgent need for biomarkers to predict response to targeted therapy and to monitor emergent drug resistance. Therapy decision for many tumour types is based on biomarkers that are evaluated at the primary tumour by the classic biopsy approach; however, classic biopsy is not allowing monitoring of primary tumours evolution during time, while repeated sampling of metastatic sites is not always possible for practical reasons. “Liquid biopsy” based on minimally-invasive blood-based tests has the potential to be a strong complement to the classic biopsy approach; Circulating Tumour Cells (CTCs) and circulating tumour DNA (ctDNA) are the major players in liquid biopsy analysis, since they can both provide an alternative approach that enables a sensitive, dynamic and specific serial tumour sampling during the course of treatment, and an early marker of response to systemic therapy [1–3]. An important requisite for liquid biopsy technologies is that CTCs or ctDNA are present in blood, and that they can be successfully isolated and analyzed. One major question for liquid biopsy technologies is whether CTCs and ctDNA composition is representative of the patient's tumour and whether they provide comparable or complementary information.

Nowadays, the detection and molecular characterization of CTCs is one of the most active and hot areas of translational cancer research [3, 4]. Therefore there is a considerable interest in CTCs research and CTCs assays development since these cells are well-defined targets for understanding tumour cell dissemination [5, 6]. The clinical importance of CTCs enumeration has been shown and is FDA-cleared for metastatic breast, colorectal and prostate cancer already a decade ago [7]. Especially in breast cancer CTCs detection and enumeration is associated with poor outcome and a number of clinical trials are now further evaluating its clinical importance [8]. Many groups so far, even by using completely different experimental approaches have clearly shown that understanding CTCs biology could be a key issue in favor of cancer patients [9].

On the other hand, the verified presence of extractable amounts of ctDNA circulating in serum and plasma of cancer patients, suggests that ctDNA carrying tumour specific alterations has a very strong potential as a liquid biopsy biomarker as well [10, 11]. The analysis of ctDNA in plasma is an extremely promising tool to identify mutations relevant to certain targeted therapies and monitor tumour evolution during disease progression [10, 11]. Moreover, ctDNA is a relatively stable biological material, much easier to isolate and analyze than CTCs. The recent identification of mutations, translocations, or copy number variations previously found in resected tumours by the classic biopsy approach in ctDNA represents a quite simple liquid biopsy approach. However even ctDNA analysis seems simpler to perform than CTCs, we must have in mind that it requires very sensitive techniques since ctDNA is just a very small percentage of cell free DNA (cfDNA) that is circulating in plasma [12, 13]. In a very limited number of breast cancer patients, it was recently shown that ctDNA provided the earliest measure of treatment response in metastatic breast cancer [14].

DNA methylation plays a fundamental role in the development and progression of many types of cancer, mainly through the inactivation of certain tumour-suppressor genes [15]. Moreover, DNA methylation is considered to be an early event in the process of cancer development and progression since tumour suppressor genes are frequently inactivated at very early stages. Detection of tumour-specific DNA methylation alterations in ctDNA could provide important information for the clinical assessment of breast cancer patients [16]. Our group has shown for the first time that tumour suppressor and metastasis suppressor genes are epigenetically silenced in CTCs isolated from peripheral blood of breast cancer patients [17]. Moreover we have recently shown that *SOX17* promoter is highly methylated in primary breast tumours, in CTCs isolated both from patients with early and metastatic breast cancer, and in corresponding ctDNA samples [18].

The primary goal of our present study was to investigate to what extent DNA isolated from CTCs and plasma is representing the primary tumour and whether is carries similar information in terms of DNA methylation biomarkers. To address this question we performed a direct comparison study of the methylation status of SRY (sex-determining region Y)-box 17 (*SOX17)*, Cystatin M (*CST6)* and Breast cancer metastasis suppressor 1 (*BRMS1)* in primary tumours, EpCAM-positive pooled CTC-fractions and ctDNA in well characterized matched clinical samples of breast cancer patients.

## RESULTS

**Figure 1:**
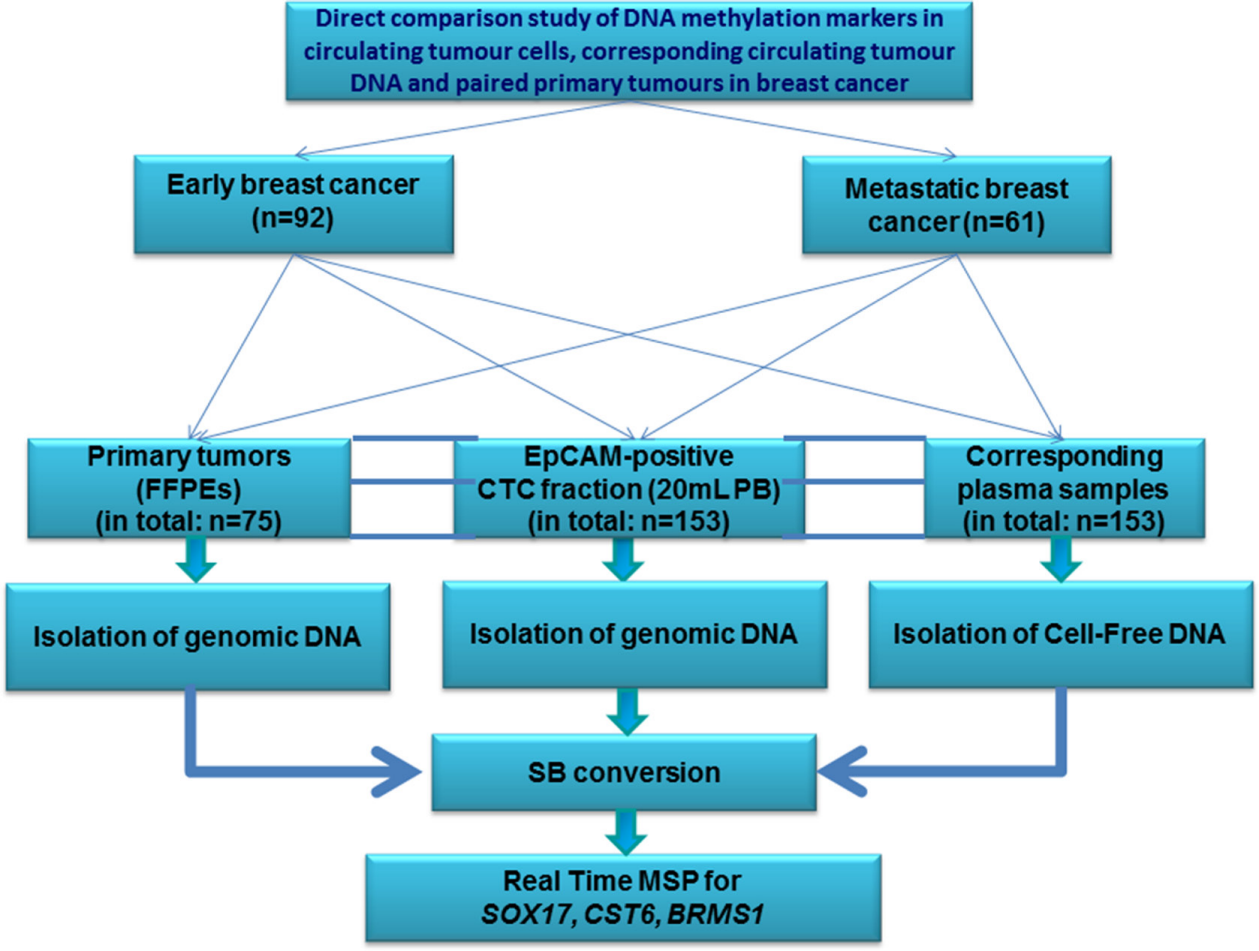
Workflow of the study

An outline of our study is shown in Figure 1.

### Analytical sensitivity and specificity of real time MSP assays

Evaluation of the analytical sensitivity and specificity of the real time MSP assays used for the evaluation of *SOX17, CST6* and *BRMS1* methylation was crucial, since ctDNA is only a fraction of cfDNA circulating in plasma, while CTCs are highly heterogeneous and only a subgroup of cells is expected to carry DNA methylated sequences. The analytical sensitivity of the developed real-time MSP assays for *SOX17, CST6* and *BRMS1* was evaluated by using the above described synthetic control mixtures. According to our results, the developed real-time MSP assays for *SOX17, CST6* and *BRMS1* could specifically and reliably detect methylated sequences when present at 0.1%. The analytical specificity of the developed real-time MSP assays for *SOX17, CST6* and *BRMS1*, was validated by initially testing all primers *in silico* and then in PCR, using SB-modified human placental gDNA (0% methylated) and unconverted DNA; no amplification of the *SOX17, CST6* and *BRMS1* promoter was observed.

### *SOX17*, *CST6* and *BRMS1* promoter methylation in primary breast tumours (FFPEs)

FFPE samples were available for 75 out of these 153 patients (Table 1). *SOX17* was detected in 26/42 (61.9%) patients diagnosed with early breast cancer and in 21/33 (63.6%) patients that developed metastasis. *CST6* promoter methylation was observed in 11/31 (35.5%) patients with early breast cancer and 16/32 (50.0%) patients with metastasis. *BRMS1* promoter methylation was detected in 19/42 (45.2%) patients with early breast cancer and 5/32 (15.6%) metastatic patients. There was no correlation between the methylation status of these genes and tumour size, number of lymph nodes, tumour grade, tumour stage, PR and ER receptors, HER2 status and age (data not shown). The diagnostic specificity of real-time MSP assays for each gene was further evaluated in non-cancerous breast tissues (mammoplasties) and fibroadenomas used as controls. *SOX17* promoter was methylated in 3/29(10.3%) non-cancerous breast tissues, and in 2/9 (22.2%) breast fibroadenomas. *CST6* promoter was methylated in 3/29 (10.3%) non-cancerous breast tissues (mammoplasties) and in 1/10 (10.0%) breast fibroadenomas. *BRMS1* promoter methylation was detected neither in 29 non-cancerous breast tissues nor in the 10 fibroadenoma samples tested.

**Table 1:** *SOX17*, *CST6* and *BRMS1* gene promoter methylation in the EpCAM positive CTC-fraction, paired ctDNA and corresponding FFPE samples

Samples	*SOX17*	*CST6*	*BRMS1*
**Early breast cancer group (*n*** **= 92)**			
EpCAM-positive CTC-fraction	19/92 (20.7%)	24/92 (26.1%)	20/92 (21.7%)
paired ctDNA	24/92 (26.1%)	33/92 (35.9%)	22/92 (23.9%)
FFPEs*	26/42 (61.9%)	11/31 (35.5%)	19/42 (45.2%)
**Metastatic breast cancer group (*n*** **= 61)**			
EpCAM-positive CTC-fraction	26/61 (42.6%)	21/61 (34.4%)	31/61 (50.8%)
paired ctDNA	22/61 (36.1%)	26/61 (42.6%)	4/61 (6.6%)
FFPEs^*^	21/33 (63.6%)	16/32 (50.0%)	5/32 (15.6%)
**Control group**			
EpCAM-positive CTC-fraction (healthy individuals)	1/23 (4.3%)	1/23 (4.3%)	2/23 (8.7%)
Plasma, cfDNA	1/49 (2.0%)	2/49 (4.1%)	2/49 (4.1%)
Fibroadenomas (FFPEs)	2/9 (22.2%)	1/10 (10.0%)	0/10 (0%)
Non-cancerous breast tissues (FFPEs - mammoplasties)	3/29(10.3%)	3/29 (10.3%)	0/29 (0%)

*: FFPEs were not available for all clinical samples studied at the peripheral blood level (CTCs and ctDNA).

### *SOX17*, *CST6* and *BRMS1* promoter methylation in DNA isolated from the EpCAM-positive CTC-fraction

We evaluated *SOX17, CST6,* and *BRMS1* promoter methylation in the EpCAM-positive CTC-fraction of 153 DNA samples isolated from 92 patients with early and 61 patients with metastatic breast cancer (Table 1). *SOX17* promoter was methylated in 19/92 (20.7%) patients with early and in 26/61 (42.6%) patients with metastatic breast cancer. *CST6* promoter was methylated in 24/92 (26.1%) patients with early, and in 21/61 (34.4%) patients with metastatic breast cancer. *BRMS1* promoter was methylated in 20/92 (21.7%) patients with early, and in 31/61 (50.8%) patients with metastatic breast cancer. According to our findings there was no correlation between the promoter methylation status of these genes in the EpCAM-positive CTC-fraction and tumour size, number of lymph nodes, tumour grade, tumour stage, the presence of progesterone (PR) and estrogen receptors (ER), HER2 status, and age (data not shown). The diagnostic specificity of real-time MSP assays for each gene was further evaluated by analyzing the EpCAM-positive CTC-fraction of 23 healthy individuals used as control group revealing that promoter methylation of *SOX17* was observed in 1/23 (4.3%), *CST6* in 1/23 (4.3%) and *BRMS1* in 2/23 (8.7%) healthy individuals.

### *SOX17*, *CST6* and *BRMS1* promoter methylation in ctDNA isolated from matched plasma samples

We further evaluated the methylation status of *SOX17, CST6,* and *BRMS1* promoter methylation in ctDNA isolated from matched plasma samples from the same 153 patients as above and 49 healthy individuals (Table 1). Promoter methylation of *SOX17* was observed in 24/92 (26.1%) patients with early breast cancer, in 22/61 (36.1%) patients with metastatic disease and in 1/49 (2.0%) healthy individuals. Promoter methylation of *CST6* was observed in 33/92 (35.9%) patients with early, 26/61 (42.6%) patients with metastatic breast cancer and 2/49 (4.1%) healthy individuals. Promoter methylation of *BRMS1* was observed in 22/92 (23.9%) patients with early and 4/61 (6.6%) patients with metastatic breast cancer and 2/49 (4.1%) healthy individuals. Finally, there was no correlation between the methylation status of these genes in ctDNA and tumour size, number of lymph nodes, tumour grade, tumour stage, the presence of PR and ER receptors, HER2 status, and age (data not shown).

### Direct comparison between *SOX17*, *CST6*, and *BRMS1* methylation in the EpCAM-positive CTC-fraction, ctDNA and corresponding primary tumours

We further directly compared the methylation status of *SOX17, CST6,* and *BRMS1* promoter in the EpCAM-positive CTC-fraction, corresponding ctDNA and paired FFPEs and investigated whether there is a direct association.

### Association between EpCAM-positive CTC-fraction and corresponding ctDNA

We found a concordance between the EpCAM-positive CTC-fraction and ctDNA in both early and metastatic breast cancer only for *SOX17* promoter methylation but not for *CST6* and *BRMS1* (Table 2). There was a clear association for *SOX17* promoter methylation both for patients with early (*P* = 0.001, 71/92, 77.2% cases) and metastatic breast cancer (*P* = 0.046, 39/61, 63.9% cases).

**Table 2:** Comparison between *SOX17*, *CST6* and *BRMS1* gene promoter methylation in the EpCAM-positive CTC-fraction and corresponding paired ctDNA samples in early (*n* = 92) and metastatic breast cancer patients (*n* = 61)

Early breast cancer group							
Gene promoter methylation	EpCAM-positive CTC-fraction	ctNA		TOTAL	*p* value	κ	% concordance
		U	M				
*SOX17*	**U**	60	13	73			
	**M**	8	11	19	***0.001***	***0.3653***	**71/92 (77.2%)**
	**TOTAL**	68	24	92			
*CST6*	**U**	44	24	68			
	**M**	15	9	24	***ns^a^***	**0.0197**	**53/92 (57.6%)**
	**TOTAL**	59	33	92			
*BRMS1*	**U**	56	16	72			
	**M**	14	6	20	***ns^a^***	**0.0751**	**62/92 (67.4%)**
	**TOTAL**	70	22	92			
**Metastatic breast cancer group**							
**Gene promoter methylation**	**EpCAM positive CTC-fraction**	**ctNA**		**TOTAL**	***p*** **value**	**κ**	**%concordance**
		**U**	**M**				
*SOX17*	**U**	26	9	35			
	**M**	13	13	26	***0.046***	**0.2478**	**39/61 (62.9%**)
	**TOTAL**	39	22	61			
*CST6*	**U**	25	15	40			
	**M**	10	11	21	***ns^a^***	**0.1408**	**36/61 (59.0%)**
	**TOTAL**	35	26	61			
*BRMS1*	**U**	28	2	30			
	**M**	29	2	31	***ns^a^***	**/**	**30/61 (49.2%)**
	**TOTAL**	57	4	61			

a: non significant.

### Association between the EpCAM-positive CTC-fraction and corresponding FFPEs

In early breast cancer we found no association between the EpCAM-positive CTC-fraction and 42 available paired primary tumours in respect to the promoter methylation status of any of the three genes studied (Table 3); concordance for *SOX17* promoter methylation was observed only in 15/42 (35.7%) cases, while the concordances were very low for *CST6,* and *BRMS1*. In the metastatic setting, there was a concordance for 21/33 (63.6%) of cases for *SOX17* promoter methylation, while there was no concordance for *CST6* and *BRMS1*.

**Table 3:** Comparison between *SOX17, CST6* and *BRMS1* gene promoter methylation in the EpCAM-positive CTC-fraction and corresponding paired FFPE samples, and in ctDNA and corresponding FFPEs in early and metastatic breast cancer patients

EpCAM-positive CTC-fraction versus corresponding paired FFPE samples: Early breast cancer group							
Gene promoter methylation	EpCAM positive CTC-fraction	FFPEs		TOTAL	*p* value	κ	% concordance
		U	M				
*SOX17*	**U**	12	23	35			
	**M**	4	3	7	***ns^a^***	**/**	**15/42 (35.7%)**
	**total**	16	26	42			
*CST6*	**U**	17	8	25			
	**M**	3	3	6	***ns^a^***	**0.1367**	**20/31 (64.5%)**
	**total**	20	11	31			
*BRMS1*	**U**	19	17	36			
	**M**	4	2	6	***ns^a^***	**/**	**21/42 (50%)**
	**total**	23	19	42			
**EpCAM-positive CTC-fraction versus corresponding paired FFPE samples:** **Metastatic breast cancer group**							
**Gene promoter methylation**	**EpCAM- positive CTC-fraction**	**FFPEs**		**TOTAL**	***p*** **value**	**κ**	**% concordance**
		**U**	**M**				
*SOX17*	**U**	8	8	16			
	**M**	4	13	17	***ns^a^***	**0.2667**	**21/33 (63.6%)**
	**total**	12	21	33			
*CST6*	**U**	10	11	21			
	**M**	6	5	11	***ns^a^***	**/**	**15/32 (46.9%)**
	**total**	16	16	32			
*BRMS1*	**U**	17	2	19			
	**M**	10	3	13	***ns^a^***	**0.1390**	**20/32 (62.5%)**
	**total**	27	5	32			
**ctDNA versus corresponding paired FFPE samples:** **Early breast cancer group**							
**Gene promoter methylation**	**ctDNA**	**FFPEs**		**TOTAL**	***p*** **value**	**κ**	**% concordance**
		**U**	**M**				
*SOX17*	**U**	11	18	29			
	**M**	5	8	13	***ns^a^***		**19/42 (45.2%)**
	**total**	16	26	42			
*CST6*	**U**	15	4	19			
	**M**	5	7	12	***ns^a^***	***w***	**22/31 (71.0%)**
	**total**	20	11	31			
*BRMS1*	**U**	20	16	36			
	**M**	3	3	6	***ns^a^***	***0.0292***	**23/42 (54.8%)**
	**total**	23	19	42			
**ctDNA versus corresponding paired FFPE samples:** **Metastatic breast cancer group**							
**Gene promoter methylation**	**ctDNA**	**FFPEs**		**TOTAL**	***p*** **value**	***κ***	**% concordance**
		**U**	**M**				
*SOX17*	**U**	8	12	20			
	**M**	4	9	13	***ns^a^***	***0.0833***	**17/33 (51.5%)**
	**total**	12	21	33			
*CST6*	**U**	11	7	18			
	**M**	5	9	14	***ns^a^***	***0.250***	**20/32 (62.5%)**
	**total**	16	16	32			
*BRMS1*	**U**	26	4	30			
	**M**	1	1	2	***ns^a^***	***0.2157***	**27/32 (84.4%)**
	**total**	27	5	32			

a: non significant.

### Association between *ctDNA* and corresponding *FFPEs*

In early breast cancer, we found no association between ctDNA and 42 available paired primary tumours in respect to the promoter methylation status of these three genes (Table 3); concordance for *SOX17* promoter methylation was observed in 19/42 (45.2%) cases while the concordances for *CST6,* and *BRMS1* were very low. In the metastatic setting, for *SOX17* there was a concordance for 17/33 (51.5%) cases and there was no concordance for *CST6,* and *BRMS1*.

### Association between *CK-19* mRNA expression and *SOX17*, *CST6* and BRMS1 promoter methylation in the EpCAM-positive CTC-fraction

**Figure 2:**
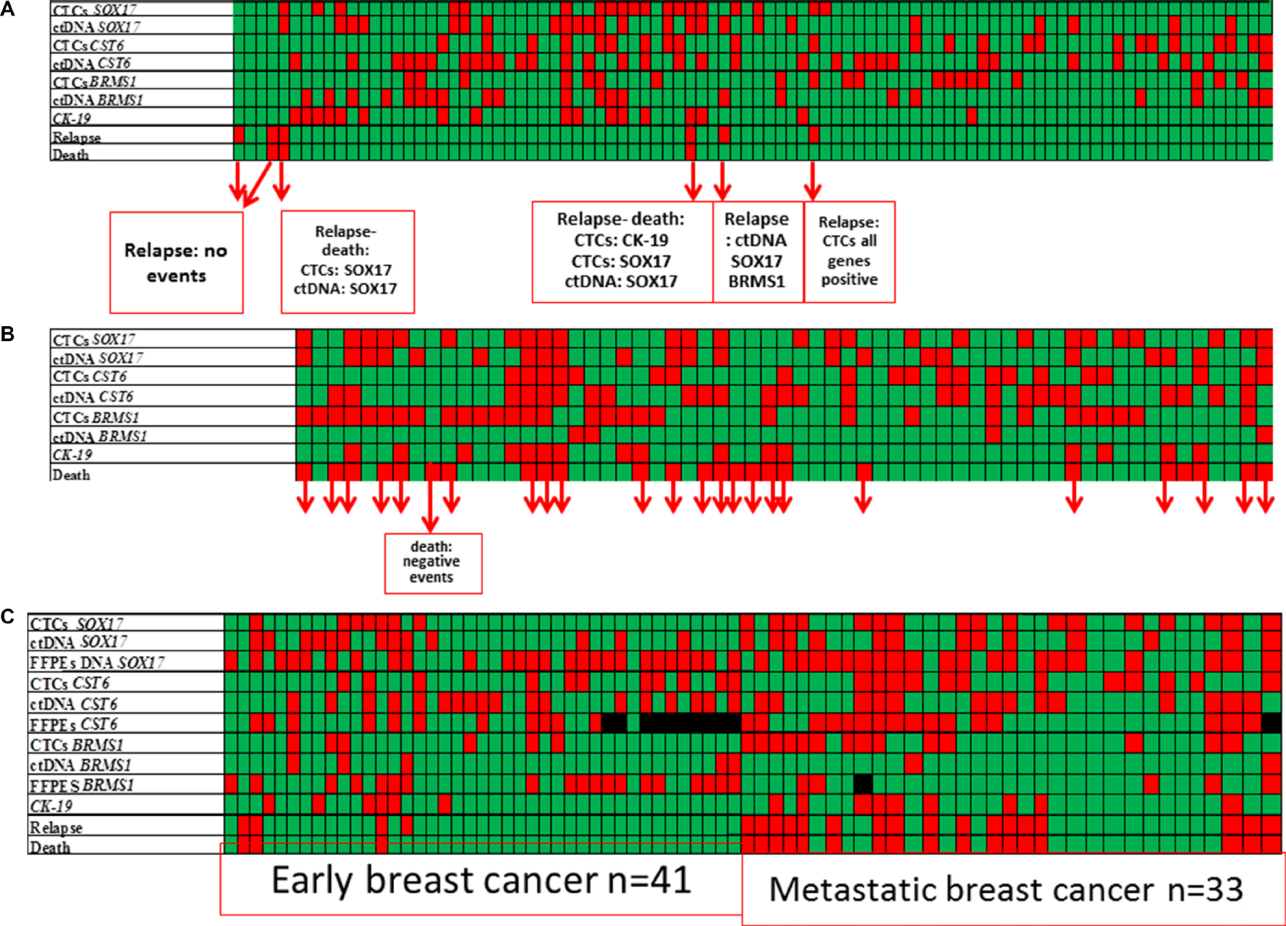
Heat map of *SOX17*, *CST6* and *BRMS1* promoter methylation and *CK-19* expression in the EpCAM-positive CTC-fraction, and ctDNA in matched samples of patients with: (**A**) operable breast cancer (*n* = 92), (**B**) verified metastasis (*n* = 61), and (**C**) all samples including available FFPEs (*n* = 75). Color code: green: non-methylated/no-relapse/alive, red: methylated/relapse/dead.

We further examined *CK-19* mRNA expression in all EpCAM-positive CTC-fractions, since we have extensively used this epithelial marker to verify the presence of CTCs (19–21). Using the same procedure, none of 60 healthy individual samples tested (0%) was found positive for *CK-19* expression. We further studied the association between *CK-19* mRNA expression and *SOX17, CST6* and *BRMS1* promoter methylation in the EpCAM- positive CTC-fraction. Results are shown in heat maps for each individual patient in all cases (Figure 2).

### Early breast cancer

In early breast cancer, *SOX17* methylation status in the EpCAM-positive CTC-fraction was found to be correlated with *CK-19* mRNA expression (*P* = 0.006). Results are shown as a heat maps for each individual patient in Figure 2A.

More specifically, 17/92 (18.5%) samples were positive for *CK-19* mRNA while 19/92 (20.6%) samples were positive for *SOX17* methylation; 8/92 (8.7%) samples were positive for both *SOX17* promoter methylation and *CK-19* mRNA and 9/92 (9.8%) samples were positive for *CK-19*, but were not carrying *SOX17* promoter methylation. It is highly remarkable that *SOX17* promoter methylation was also identified in the EpCAM-positive CTC-fraction of 11 patients who were negative for *CK-19* mRNA expression. However, in the same group *CST6* and *BRMS1* methylation status was not correlated with *CK-19* mRNA expression. More specifically, only 4/92 (4.3%) samples were positive for both *CST6* promoter methylation and *CK-19* mRNA expression, 13/92 (14.1%) samples were positive for *CK-19*, but not carrying *CST6* promoter methylation, while *CST6* promoter methylation was identified in the EpCAM-positive CTC-fraction of 20 patients who were negative for *CK-19* mRNA. In the same group only 2/92 (2.1%) samples were positive for both *BRMS1* promoter methylation and *CK-19* mRNA expression, 16/92 (17.4%) samples were positive for *CK-19* but not carrying *BRMS1* promoter methylation, while *BRMS1* promoter methylation was identified in 17 patients who were negative for *CK-19* mRNA expression.

### Metastatic disease

In this group 6/61 (26.2%) samples were found positive for *CK-19* expression in the EpCAM-positive CTC-fraction. Results are shown as a heat maps for each individual patient in Figure 2B. *SOX17, CST6* and *BRMS1* promoter methylation were not individually correlated with *CK-19* expression. However, in the EpCAM-positive CTC-fraction of the 45 patients who were negative for *CK-19* mRNA expression, *SOX17* promoter methylation was identified in 18/45 (40.0%), *CST6* promoter methylation in 14/45 (31.1%) and *BRMS1* promoter methylation in 20/45 (44.4%).

### Association between *CK-19* mRNA expression and *SOX17*, *CST6* and *BRMS1* promoter methylation in corresponding ctDNA

We further studied the association between *CK-19* mRNA expression and *SOX17, CST6* and *BRMS1* promoter methylation in corresponding ctDNA. Results are shown in heat maps for each individual patient in all cases (Figure 2).

### Early breast cancer

In this group, there was a statistically significant association between DNA methylation of one or more of these genes in ctDNA and *CK-19* expression in the corresponding EpCAM-positive CTC-fraction (*P* = 0.014). Results are shown as a heat maps for each individual patient in Figure 2A. In 58/92 (63.0%) of patients at least one of the genes studied was found methylated in ctDNA. There were 32/92 (34.8%) cases where all markers were negative, 15/92 (16.3%) cases positive for both DNA methylation in ctDNA and *CK-19* mRNA expression, while there were only 2/92 (2.2%) cases positive for *CK-19* and negative for *SOX17, CST6* and *BRMS1* promoter methylation. It is highly remarkable that DNA methylation was identified in ctDNA of 43 patients who were negative for *CK-19* mRNA expression.

### Metastatic disease

Results are shown as a heat maps for each individual patient in Figure 2B. In this group, *SOX17* promoter methylation in ctDNA was highly correlated with *CK-19* expression (*P* = 0.040). *CST6* and *BRMS1* promoter methylation in ctDNA were not individually correlated with *CK-19* expression. However samples that were found to have at least two gene promoters methylated in ctDNA were also *CK-19* mRNA positive (*P* = 0.021).

### Association of *SOX17*, *CST6*, and *BRMS1* promoter methylation in CTC, ctDNA and corresponding primary tumours with clinical outcome

We further evaluated whether there is any association between *SOX17, CST6,* and *BRMS1* promoter methylation in CTC, ctDNA and corresponding primary tumours and the clinical outcome of patients. Results are shown as a heat maps for each individual patient in Figure 2.

### Early breast cancer group

**Figure 3:**
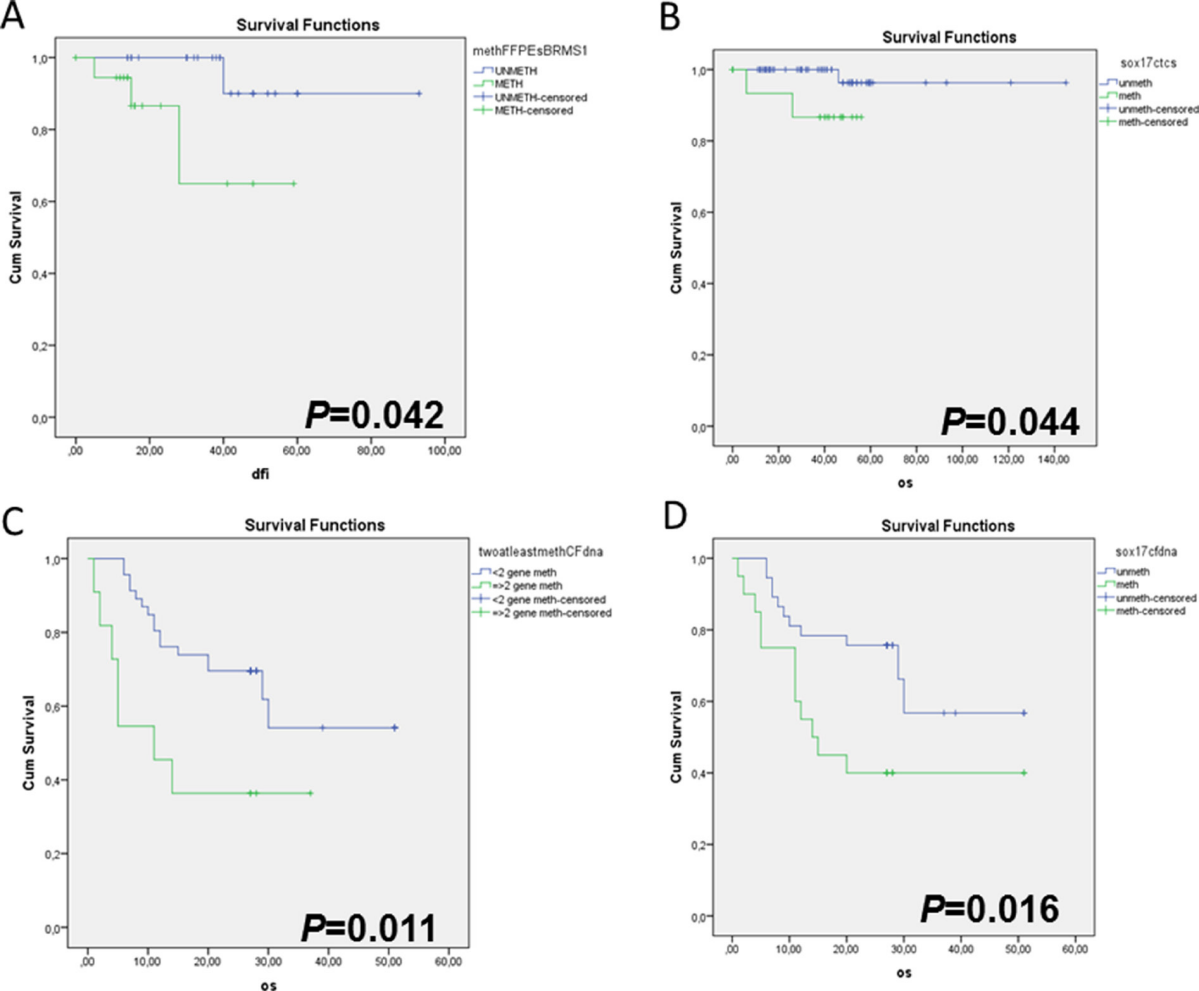
Kaplan-Meier estimates of (**A**) Disease-free interval (DFI) in months for early breast cancer patients in respect to *BRMS1* promoter methylation status in FFPEs (*P* = 0.042), (**B**) Overall survival (OS) in months for early breast cancer patients with clinically confirmed metastasis, in respect to *SOX17* promoter methylation status in CTCs (*P* = 0.044), (**C**) Overall survival (OS) in months for breast cancer patients with clinically confirmed metastasis, in respect to the methylation of at least two genes in ctDNA (*P* = 0.011), (**D**) Overall survival (OS) in months for breast cancer patients with clinically confirmed metastasis, in respect to *SOX17* promoter methylation status in ctDNA (*P* = 0.016).

After a median follow-up period of 40 months (range 6–121), 6/92 (6.5%) patients with early breast cancer relapsed and 3/92 (3.3%) of them died as a consequence of disease progression. In 4/6 patients that relapsed *SOX17* gene promoter was found highly methylated in the primary tumour, in the corresponding CTC-fraction and in ctDNA (Figure 2A). It is worth mentioning that in one patient that relapsed very early, her blood specimen was found methylated in the EpCAM-positive CTC-fraction, for all genes tested, while it was *CK-19* negative. The direct comparison study of *SOX17*, *CST6* and *BRMS1* promoter methylation in corresponding FFPEs (*n* =41) is shown in Figure 2C. Kaplan–Meier estimates of the cumulative Disease Free Interval (DFI) were significantly different in favor of patients with non-methylated *BRMS1* promoter (Figure 3A; *Ρ* = 0.042), as we have previously reported for a different cohort of breast cancer patients (27). Concerning CTCs, patients with non-methylated *SOX17* promoter in the EpCAM-positive CTC-fraction had a significantly better median overall survival (OS) compared to patients with methylated *SOX17* promoter (Figure 3B; *P* = 0.044).

### Metastatic breast cancer group

During the follow up, 25/61 (40.9%) patients with metastatic disease died as a consequence of disease progression. The direct comparison study of *SOX17*, *CST6* and *BRMS1* promoter methylation in corresponding FFPEs (*n* = 33) is shown in Figure 2C. In this group, the incidence of deaths was higher when at least two gene promoters were methylated in ctDNA in respect to patients where one or none gene promoter was methylated (Figure 3C; *P* = 0.011). It is interesting to note that the Kaplan–Meier estimates of the OS were significantly different in favor of patients with non-methylated *SOX17* promoter (Figure 3D; *P* = 0.016).

## DISCUSSION

In the present study we performed for the first time a direct comparison study of the DNA methylation status of *SOX17, CST6* and *BRMS1* in primary tumours, corresponding plasma ctDNA and in matched cell pools of EpCAM-positive CTCs, in order to investigate whether the tumour's “liquid phase” reflects the status of the primary tumour. We also wanted to evaluate to what extend these markers yield complementary or additional information about prognosis and disease progression. By using specimens derived from the same patients and identical blood draws, and by applying identical and analytically validated methodologies for the MSP assays used, we minimized most pre-analytical parameters that could affect this comparison. The procedure that we have followed for CTCs enrichment and analysis in the present study has been validated in previous studies and has been compared with commercially available molecular assays [20–23].

DNA methylation of these three gene promoters has been extensively studied so far mainly in primary tissues of various types of cancer. We and others have already shown that detection of *CST6* promoter methylation in primary tissues provides important prognostic information in patients with operable breast cancer [24, 25]. Furthermore, we reported for the first time that *CST6* promoter is methylated in ctDNA and is promising as a highly specific tumour biomarker for early breast cancer patients since it was not detected in plasma of healthy individuals [26].

We have recently shown that *BRMS1* promoter methylation in the primary tumour was associated with poor DFS while our study on BRMS1 protein expression by immunofluorescence revealed CTCs heterogeneity even in the same patient [22]. BRMS1 is a predominantly nuclear protein that differentially regulates expression of multiple genes, leading to suppression of metastasis without blocking orthotopic tumour growth [27, 28]. *BRMS1* mRNA expression has been shown to be markedly reduced in melanoma, breast cancer, ovarian cancer and non-small cell lung cancer cell lines, while stable overexpression of BRMS1 in these cell lines significantly inhibited their metastatic potential [29–31].

SOX17 plays a critical role in the regulation of development and stem/precursor cell function, at least partly through repression of the canonical Wnt/β-catenin signaling pathway [32, 33]. We have shown already in a limited number of patients that *SOX17* promoter is highly methylated in CTCs isolated from patients with breast cancer [17], and in corresponding ctDNA samples [17, 18].

According to our findings, there was no concordance between the EpCAM-positive CTC-fraction and paired primary tumours in respect to the promoter methylation status of any of the genes studied, neither in early, nor in the metastatic setting. A lack of concordance was also found for the same DNA methylation markers between ctDNA and matched primary tumours. A possible explanation for this finding could be based on tumour heterogeneity and rapid evolution through time, indicating that the liquid biopsy is reflecting the actual phase of tumour evolution. It is now clear that CTCs are not only rare but heterogeneous at the same time, even within the same patient [34, 35]. This could partially explain the differences seen in our study.

There are very few studies so far comparing DNA methylation markers in primary tumours, CTCs and paired ctDNA samples [17, 18, 22]. In all these previous studies we have already shown that DNA methylation markers can be detected in EpCAM-positive CTC-fractions but not in the primary tumour, and that EpCAM-positive CTC-fractions and ctDNA do not give identical but highly correlated information. This is the first study where so many samples are analyzed in the primary tumour setting in EpCAM-positive CTCs and in corresponding plasma using the same blood draw, identical methodologies and three genes. A possible explanation for the discrepancies detected between primary tumours, CTCs and ctDNA could be that in the primary tumours the amount of methylated cancer cells could be a small subgroup between millions non-methylated cancer cells. Because of the heterogeneity of the tumour tissue, the tumour tissue biopsy section may contain cells that are not representative for the entire cancer volume. As a result, the methylation information of the whole tissue is more difficult to interpret because we need samples from different sites of the cancer tissue in order to characterize the whole volume. Methylated DNA sequences detected in ctDNA can be released from necrotic and apoptotic cells, while methylated DNA sequences detected in the EpCAM positive CTC-fractions are indicative of an active-metastasis condition.

Our present data indicate a direct association between *SOX17* promoter methylation in CTCs and ctDNA in patients with breast cancer. We report for the first time that in the EpCAM-positive CTC-fraction, *SOX17* promoter methylation was associated with *CK-19* mRNA expression both in patients with early and metastatic breast cancer. In addition, our findings revealed that methylation of *SOX17* promoter in ctDNA is associated with unfavorable prognosis in patients with metastatic breast cancer and that in patients with early breast cancer, methylation of *SOX17* promoter in the EpCAM-positive CTC-fraction is associated with decreased DFI. We did not find any association between the methylation status of *CST6* and *BRMS1* in CTCs, ctDNA and corresponding primary tumours. This could be attributed to tumour evolution over time and to the Epithelial Mesenchymal Transition (EMT) phase of CTCs [36].

In the EpCAM-positive CTC-fraction, we found that all these three genes were methylated at significantly higher percentages in the metastatic setting than in early stages of the disease. This could be explained by the higher tumour load in the metastatic setting. It is also worth mentioning that in plasma the number of samples found positive for *SOX17* and *CST6* promoter methylation were higher than in the EpCAM-positive CTC-fraction while non-specific methylation was detected at even lower percentages in all cases. This could possibly indicate a release of methylated DNA from apoptotic cells as well. Non-specific methylation events were detected at a very low percentage in all our control samples.

It is also interesting to note that in most early breast cancer patients that relapsed early, *SOX17* gene promoter was found highly methylated in the primary tumour, in the EpCAM-positive CTC-fraction and in ctDNA. It is also worth mentioning that the blood specimen of one early breast cancer patient that relapsed very early was found methylated in the EpCAM-positive CTC-fraction, for all genes tested, while it was *CK-19* negative. We plan to verify this finding in the future by analysing a larger number of patients. It is also remarkable to note that samples found positive for *SOX17*, *CST6* and *BRMS1* promoter methylation in the EpCAM-positive CTC-fraction were negative for *CK-19* mRNA expression. If CTCs positivity would be based only on *CK-19* expression these samples would have been characterized as CTC-negative. This indicates the importance of multi-parametric testing in CTC analysis.

In the metastatic setting, much higher detection rates for CK-19 are usually expected, as we have previously shown using the same RT-qPCR assay [19]. However, the lower percentages found for CK-19 in the present study can be explained, because only samples for which we had available primary tumours and corresponding plasma for ctDNA isolation were selected for analysis.

Molecular characterization of single CTCs combined with massive parallel sequencing (MPS) technologies performed both in single CTC and paired plasma could elucidate further the association between CTCs and ctDNA [37, 38]. Very recent studies have clearly shown that the isolation, *ex vivo* culture, and characterization of CTCs is a very promising approach to monitor continuously and in a non-invasive way the changing patterns of drug susceptibility in individual patients as their tumours acquire new mutations [39, 40]. However, the processing of blood specimens for CTC analysis is complicated and time consuming. By comparison, ctDNA analysis has recently gained a lot of attendance since it has the potential to be convenient and relatively simple to process in a short time period [41, 42]. Recently the feasibility and potential utility of plasma ctDNA as an alternative to metastatic biopsies for mutational analysis in breast cancer using NGS has been shown [43]. However in this study, discordances between primary tumours and plasma were also reported. Recent results demonstrate also that exome sequencing on cfDNA is a very powerful tool for disease monitoring of metastatic cancers [44].

Concerning the comparison between CTCs and ctDNA, the most important parameter is correlation of the biomarker under evaluation with the clinical outcome. As an example, in a very recent study, Madic et al. used the high prevalence of TP53 mutations in triple negative breast cancer (TNBC) to compare ctDNA and CTCs detection rates and evaluate their prognostic value in metastatic TNBC patients [45]. They used different markers and different methodologies to compare ctDNA and CTCs detection rates, the presence of TP53 for ctDNA as verified by NGS, and the CellSearch^TM^ FDA cleared system for the enumeration of CTC. They report an absence of prognostic impact of baseline ctDNA level suggesting that mechanisms of ctDNA release in metastatic TNBC may involve, beyond tumour burden, biological features that do not dramatically affect patient outcome [45].

CTCs molecular analysis when compared to ctDNA and exosomes as a liquid biopsy approach has the clear advantage that its prognostic significance has already been shown through numerous studies [5–9], and that it addresses viable cells that are not simply carriers of individual tumour biomarkers. Moreover, CTCs represent live tumour cell entities that can give information on emerging tumour sub-clones with altered mutational and drug sensitivity profiles and can be targetable by specific drugs. From this point of view, CTCs molecular characterization can offer an essential component of personalized cancer treatment [4].

In conclusion our findings indicate a direct association only between *SOX17* promoter methylation in CTCs and ctDNA both in patients with early breast cancer and in patients with metastatic disease but not for the other genes studied. We could not identify a direct association between the methylation status of these genes in primary tumours CTCs and ctDNA, possibly because tumours are evolving and the liquid biopsy is reflecting the actual state of tumour evolution. Combination of DNA methylation analysis of tumour suppressor and metastasis suppressor genes in EpCAM-positive CTCs and matched ctDNA provides significant prognostic value. We plan to extend this direct comparison study and verify these findings in larger cohorts and for a wide range of gene promoters, using Massive Parallel Sequencing technologies. We will evaluate the clinical significance of these findings in an independent study, in respect to the clinical characteristics of the patients and additional markers already studied by our group on CTCs (methylation, mutations, miRNAs and gene expression) in a large number of patients with a known clinical outcome and a longer follow up.

## MATERIALS AND METHODS

### Patients

We directly compared *SOX17, CST6* and *BRMS1* promoter methylation in ctDNA and in the EpCAM-positive CTC-fraction of 153 breast cancer patients; 92 with early and 61 with metastatic breast cancer. Peripheral blood was isolated from all these patients for CTCs and ctDNA analysis. In early breast cancer cases peripheral blood was collected at least two weeks after surgery and before the initiation of adjuvant chemotherapy, while in the metastasis group samples were collected before the initiation of first line treatment. For 75 of these patients the corresponding FFPEs samples from the primary tumours were available. In all cases the primary tumor of patients that relapsed after several months or years had been analysed. More specifically we analysed: a) EpCAM-positive CTC-fraction samples isolated from peripheral blood of all patients (*n* = 153); for all these samples, information on the expression of *CK-19* in the EpCAM-positive CTC-fraction was also available, b) ctDNA samples isolated from corresponding plasma of the same patients during the same venepuncture (*n* = 153) and c) 75 corresponding primary breast cancer formalin-fixed paraffin-embedded tissues (FFPE) (42 and 33 from patients with early and metastatic breast cancer, respectively). As a control population, peripheral blood was collected from 60 healthy individuals, while 29 non-cancerous breast tissues (mammoplasties) and 10 breast fibroadenomas were used as non-cancerous breast tissue controls. All study participants signed an informed consent form to participate in the study, which was approved by the ethics and scientific committees of our institutions.

### DNA isolation from FFPEs

Tissue sections of 10 μm containing 80% tumour cells were used for DNA extraction. DNA 100% methylated (DNA methylation standard, Zymo Research) was used as a positive control in MSP for the detection of *SOX17*, *CST6* and *BRMS1* promoter methylation. Genomic DNA (gDNA) from FFPEs was isolated using the QIAamp DNA FFPE Tissue Kit (Qiagen, Germany). DNA concentration was determined in the Nanodrop ND-1000 spectrophotometer (Nanodrop Technologies, USA).

### Isolation of the EpCAM-positive CTC-fraction

The EpCAM-positive CTC-fraction was isolated from 20 mL peripheral blood as previously described [17, 18, 46]. All DNA isolation and handling steps took place in a dedicated area and in a laminal flow hood. gDNA was extracted from the EpCAM-positive CTC-fraction as previously described [17, 18]. Total RNA isolation from the EpCAM-positive CTC-fraction was performed using Trizol (Invitrogen, USA) as previously described [46]. All RNA preparation and handling steps took place in a dedicated area in a laminar flow hood, under RNase-free conditions. mRNA was isolated from the total RNA by use of the Dynabeads mRNA purification Kit (Invitrogen, USA) according to the manufacturer's instructions. cDNA synthesis was performed using the High-Capacity RNA-to-cDNA kit (Applied Biosystems, USA) and was used for *CK-19* expression studies in CTCs as previously described [46].

### Isolation of ctDNA from plasma

Peripheral blood in EDTA was collected and processed immediately for plasma isolation. All samples were centrifuged at 1600 g (10 min), and plasma was carefully transferred into 2 mL tubes and stored at −20°C until ctDNA isolation. The High Pure Viral nucleic acid kit (Roche Diagnostics) was used to extract ctDNA from plasma (200 μL) as previously described [17, 18].

### Sodium bisulfite conversion

Preparation of sodium bisulfite (SB) conversion reaction was performed in a laminar flow hood, in a dedicated separate room. For all clinical samples, before proceeding to SB conversion and MSP reaction steps, the integrity of isolated gDNA was assessed by amplifying exon 20 of the BRCA1 gene as previously described [47]. gDNA extracted from both isolated EpCAM-positive CTC-fractions and plasma and gDNA from FFPEs was SB-modified using the EZ DNA methylation–Gold kit (Zymo Research, USA) as previously described [17, 18]. In each set of SB reactions, deionized water and DNA 100% methylated (DNA methylation standard, Zymo Research) were included as negative and positive control, respectively.

### Real time methylation specific PCR (MSP)

For high throughput analysis of our samples we designed highly specific and sensitive real Time MSP assays for each gene of interest. All experiments were performed in the LightCycler 2.0 (*IVD* instrument, Roche, Germany). All oligonucleotides were *de-novo in-silico* designed for each gene by using the PrimerPremier 5 software (Premier Biosoft International, USA), and synthesized by IDT (Intergraded DNA technologies, USA) [26, 48]. For each gene, primer pairs and LNA probes are given in Supplementary Table 1. Each reaction was performed in a total volume of 10 μL, and 1 μL of SB-converted DNA was added to a 9 μL reaction mixture [17, 18, 22].

### Quality control of real time MSP

To verify that we could specifically detect only the targeted methylated sequences, in each MSP reaction we used the following controls: a) gDNA not submitted to SB-conversion (unconverted DNA) and placental DNA submitted to SB-conversion (placental converted DNA, 0% methylated), were included as negative controls and b) SB-converted DNA from the DNA methylation standard (100%) were included in every run as positive control. To exclude false negative results, all SB-converted samples found negative by real-time MSP, were checked for DNA quality by using a specifically designed primer set that equally amplifies in the same genomic region both methylated and non-methylated SB-converted target sequences.

To evaluate the analytical sensitivity of the developed real-time MSPs we prepared synthetic control mixtures by serial dilutions of SB-converted DNA control samples (0% and 100% methylated). More specifically the SB-converted DNA methylation standard (100%) was spiked in SB-converted placental DNA (0% methylated) in a concentration range of 0%, 1%, 25%, and 50%. For the evaluation of analytical sensitivity, 1 μL of these synthetic samples were used in the Real- Time MSP reactions.

### Statistical analysis

We used the χ^2^ test of independence for data analysis and for the evaluation of the significance of differences between groups. We used the Fisher exact test and Cohen's kappa coefficient, for the evaluation of agreement between methylation of each gene in respect to its presence in CTCs and ctDNA and in respect to *CK-19* gene expression [49]. Correlations between methylation status and clinico-pathological features of the patients were assessed by using the Chi-square test. Disease free interval (DFI), Progression Free Survival (PFS) and overall survival (OS) curves were calculated by using the Kaplan Meier method and comparisons were performed using the long rank test. *P* values < 0.05 were considered statistically significant. Statistical analysis was performed by using the SPSS Statistics 22.0 Windows program (SPSS Inc., Chicago, IL).

## SUPPLEMENTARY TABLE


