# Supplementary material for: Direct comparison study of DNA methylation markers in EpCAM-positive circulating tumour cells, corresponding circulating tumour DNA, and paired primary tumours in breast cancer
Source: Oncotarget. 2017 Jun 27;8(42):72054–68. doi: 10.18632/oncotarget.18679 (PMC5641111; doi:10.18632/oncotarget.18679)
Supplement: Supplementary file 1 [file oncotarget-08-72054-s001.pdf]

Direct comparison study of DNA methylation markers in EpCAM-positive circulating tumour cells, corresponding circulating tumour DNA, and paired primary tumours in breast cancer

SUPPLEMENTARY MATERIALS

Supplementary Table 1: Oligonucleotide sequences of *SOX17*, *CST6*, and *BRMS1* MSP-primers and LNA probes used in this study

Primers and LNA probes	Sequence 5′-3′ direction	PCR (bp)
SOX17		
MSP Forward	-GTTGCGTTAGTCGTTTGCGTT-	76
MSP Reverse	-AACGAATCCCGTATCCGACG-	
LNA Probe	-F-AGTTTATATTATGAAAGbCGTTTATCGGT-Q	
GenBank:	NT_008183	
CST6		
MSP Forward	-TCGAGTTTCGTTTTAGTTTTAGGTC-	134
MSP Reverse	-CAT AACCGTCAATACCGTCG-	
LNA Probe	-F-TAGCGGGTAAAAGTTGCGCGGTCGTAAGTT-Q	
GenBank:	NT 033903	
BRMS1		
MSP Forward	-GTAGATGTTTTACGTTATTCGG-	119
MSP Reverse	-CCTCCTACCCGTACAATCCGA-	
LNA Probe	-F-ACAAATAAAA+C+A+A+CT+A+C+AAC—Q	
GenBank:	NM_006577	
